# Transmission sexuelle de la strongyloïdose chez des hommes ayant des relations sexuelles avec des hommes (HSH) à Paris

**DOI:** 10.48327/mtsi.v5i1.2025.660

**Published:** 2025-03-05

**Authors:** Michel DEVELOUX, Martin SIGUIER, Christophe HENNEQUIN, Gilles PIALOUX

**Affiliations:** 1 Service de maladies infectieuses et tropicales, Hôpital Tenon, APHP, Paris, France; 2 Laboratoire de parasitologie-mycologie, Hôpital Saint-Antoine, Paris, France; 3 Sorbonne Université, Paris, France

**Keywords:** Strongyloïdose, HSH, transmission sexuelle, Anulingus, Coprophagie, Paris, France, Europe, Strongyloidiasis, MSM, Sexual transmission, Rimming, Coprophagy, Paris, France, Europe

## Abstract

**Introduction:**

La transmission sexuelle des parasites intestinaux est signalée depuis longtemps, avant la pandémie du VIH. Elle concerne surtout des protozoaires. Plus rarement il s'agit d'helminthes : *Strongyloides stercoralis, Enterobius vermicularis.* Ce type de transmission s'observe presque exclusivement chez des hommes ayant des relations sexuelles avec des hommes (HSH) en raison de contacts sexuels directs oraux-génitaux-anaux.

**Observations:**

Nous présentons quatre cas de strongyloïdose transmise sexuellement chez des HSH.

**Discussion:**

Plusieurs facteurs associés ont été retrouvés : infection VIH, épisodes récurrents d'autres infections sexuellement transmissibles, ou des facteurs de risques : relations sexuelles avec des hommes originaires de régions endémiques pour *S. stercoralis*, anulingus, *chemsex*, pratiques scatologiques. Chez les HSH, une strongyloïdose doit être évoquée et recherchée, devant une éosinophilie pouvant être fluctuante, accompagnée ou non de signes digestifs et ou cutanés, même en l'absence de séjour en zone tropicale.

**Conclusion:**

L'interrogatoire de ces patients HSH doit être minutieux, faisant préciser en détail leurs comportements sexuels conduisant à des conseils prophylactiques adaptés tel que des rapports oraux-génitaux-anaux non protégés.

## Abstract (en)

**Introduction:**

The sexual transmission of intestinal parasites has been reported for a long time, even before the HIV pandemic. It mainly involves protozoa. More rarely, helminths are involved: *Strongyloides stercoralis, Enterobius vermicularis.* This type of transmission is almost exclusively observed in men who have sex with men (MSM) through direct oral-genital-anal sexual contact.

**Observations:**

We present four cases of sexually transmitted strongyloidiasis in MSM.

**Discussion:**

Several associated factors were found: HIV infection, recurrent episodes of other sexually transmitted infections, or risk factors: sexual relations with men from regions endemic for *S. stercoralis*, rimming, chemsex, scatological practices. In MSM, strongyloidiasis must be considered and investigated in the presence of eosinophilia, which may be fluctuating, with or without digestive and/or cutaneous signs, even in the absence of a stay in a tropical zone.

**Conclusion:**

These MSM patients must be thoroughly questioned, with details of their sexual behavior leading to appropriate prophylactic advice, such as unprotected oral-genital-anal intercourse.

## Introduction

La transmission sexuelle de parasites intestinaux chez les hommes ayant des relations sexuelles avec des hommes (HSH) est signalée depuis longtemps [12, 14]. Il s'agit majoritairement de protozoaires *(Entamoeba histolytica* et *Giardia intestinalis).* La pratique de l'anulingus qui facilite la transmission fæco-orale des parasites entériques est présentée comme le principal facteur de risque prédicteur de ce type de transmission. La strongyloïdose a été très rarement décrite dans ce contexte et ne figure pas dans les revues générales récentes portant sur les Infections sexuellement transmissibles (IST) émergentes ou ré-émergentes [4, 15]. Nous présentons four cas d'infection à *Strongyloides stercoralis* chez des HSH en rapport avec certaines pratiques sexuelles chez des sujets vivant en France métropolitaine et n'ayant pas voyagé en zone d'endémie.

## Patients

### Observation 1

Un homme de 36 ans a consulté en 2012 au département de maladies infectieuses et tropicales de l'hôpital Tenon à Paris pour douleurs péri-ombilicales et hyperéosinophilie à 5 720/m^3^. Il est HSH, son infection VIH a été découverte six ans auparavant. Il est traité par truvada/prezista/norvir, la charge virale était indétectable et les CD4 étaient à 358/mm3. Le patient avait de nombreux antécédents d’IST : plusieurs épisodes de syphilis, une hépatite C et des condylomes génitaux.

Les symptômes ont débuté 10 jours avant la consultation associant douleurs abdominales et diarrhée à début brutal. Cinq jours après apparaissait une éruption urticarienne sur les fesses, disparaissant au bout de six jours.

Des larves de S. *stercoralis* étaient retrouvées à l'examen direct des selles ainsi que par concentration et méthode de Baermann. La sérologie de la strongyloïdose était négative ainsi que celle des autres helminthiases. Le patient était traité par ivermectine 200 pg/kg pendant deux jours. Les signes cliniques ont été rapidement résolutifs. Un mois après, l'hyperéosinophilie avait disparu et l'examen des selles était négatif.

Le patient avait voyagé au Maroc six ans auparavant, seule fois où il avait quitté la France. Il avait un partenaire régulier dont les recherches parasitologiques étaient négatives. Il fréquentait un sauna gay où il avait de nombreux rapports sexuels non protégés en dehors du risque VIH (charge virale indétectable) avec des hommes dont certains étaient originaires de zones endémiques pour la strongyloïdose. Il n'a pas été possible de proposer un questionnaire approfondi de ses pratiques sexuelles.

### Observation 2

Un homme de 64 ans ayant une infection VIH connue était hospitalisé en 2018 pour diarrhée depuis un mois, avec douleurs abdominales, prurit généralisé et éosinophilie élevée (9 330/mm^3^). Il se décrit comme bisexuel. L'infection VIH avait été diagnostiquée 12 ans auparavant, traitée par abacavir/dolutegravir/lamivudine (charge virale indétectable, CD4 : 779/µl). Il avait un suivi régulier avec une numération formule sanguine deux fois par an. Aucune hyperéosinophilie n'avait été détectée avant l’épisode diarrhéique. Dans les antécédents on relevait de nombreuses infections sexuellement transmissibles : *Chlamydia trachomatis*, hépatite A et C. On retrouvait la notion d'un court séjour en Afrique subsaharienne quatre ans avant l'hospitalisation. L'hyperéosinophilie était confirmée par un deuxième prélèvement. La sérologie de la strongyloïdose était positive, des larves étaient retrouvées uniquement à la méthode d'extraction de Baermann. Il a reçu deux doses d'ivermectine (200 µg/kg) à un jour d'intervalle. Les signes cliniques ont été rapidement résolutifs. Un mois après, l’éosinophilie s’était normalisée et l'examen parasitologique des selles était négatif. Il n'a pas été possible de proposer un questionnaire approfondi de ses pratiques sexuelles.

### Observation 3

Un HSH de 52 ans suivi dans le département des maladies infectieuses pour infection VIH est adressé en consultation en 2018 pour bilan d'une hyperéosinophilie (2 663/mm^3^) découverte fortuitement un an auparavant et confirmée six (1 664/mm^3^) et douze mois (1 840/mm^3^) après. L'infection VIH avait été diagnostiquée 19 ans plus tôt et il suivait un traitement antirétroviral associant dolutégravir/abacavir/lamivudine (charge virale indétectable, CD4 > 900/µl). Ses antécédents étaient marqués par plusieurs épisodes d’IST : chancre syphilitique génital, gonococcie pharyngée, hépatite C spontanément résolutive. Il n'avait jamais quitté la France, vivait avec un compagnon mais avait d'autres partenaires. Il avait des relations régulières non protégées au sein d'un « *group sex*» avec 12 à 14 partenaires par mois. Le groupe pratiquait l'anulingus et avait recours au *chemsex* (pratique associant rapport sexuel et prise de drogue). Depuis deux ans, deux migrants originaires d’Afrique subsaharienne avaient rejoint le groupe. La sérologie de la strongyloïdose du patient était positive et des larves étaient retrouvées à l'examen direct et à la concentration par formol-éther. Un traitement unique par ivermectine se révélait insuffisant et la guérison était obtenue par la même dose 2 jours de suite. Les examens parasitologiques étaient négatifs chez le compagnon, les deux personnes migrantes d’Afrique subsaharienne n'ont pas souhaité se faire dépister. En reprenant l'interrogatoire sur les pratiques sexuelles de ce patient, nous avons retrouvé, outre l'anulingus, des pratiques de *fistfucking* insertif mais non réceptif sans gant et des pratiques de scatophilie avec coprophagie réceptive.

### Observation 4

Un homme de 52 ans, HSH, ayant une infection VIH, était hospitalisé pour exploration d'une hyperéosinophilie asymptomatique (2 673/mm^3^) découverte lors d'un bilan régulier de son infection, confirmée par un contrôle deux mois après (1 533/mm^3^). Il n'avait jamais voyagé en dehors de la France métropolitaine. L'infection par le VIH avait été découverte 28 ans auparavant. Il était traité par truvada et isentress (charge virale négative, CD4 : 861). Plusieurs infections sexuellement transmissibles étaient notées dans ses antécédents : syphilis secondaire, gonorrhée anorectale, condylomes génitaux. Il avait de nombreux partenaires et pratiquait le *chemsex.* L'examen clinique était normal. Des larves de *Strongyloides stercoralis* étaient retrouvées à l'examen direct et aux méthodes de concentration, la sérologie était positive. Il a été traité avec succès par ivermectine. En reprenant l'interrogatoire sur ses pratiques sexuelles, nous avons retrouvé chez ce patient, outre l'anulingus, un épisode unique de pratiques de scatophilie avec coprophagie réceptive, après une rencontre sur un site HSH, deux mois avant le dépistage de l'hyperéosinophilie.

## Discussion

Les infections entériques à transmission sexuelle, favorisées par certaines pratiques, ont été rapportées chez des HSH [4, 15]. Elles sont bactériennes, virales ou parasitaires. Les conséquences peuvent être préoccupantes comme en témoigne la diffusion de shigelloses multi-résistantes dans différents continents [8]. La transmission par voie sexuelle de parasites entériques chez les HSH est connue depuis longtemps, avant l'apparition de VIH/sida [12, 14]. Il s'agit avant tout de protozoaires *(Entamoeba histolytica, Giardia intestinalis, Cryptosporidium* sp.) [1, 4, 14, 15]. La transmission d'helminthes *(Enterobius vermi-cularis, Strongyloides stercoralis)* est plus rare [7, 11]. Dans une étude menée dans une clinique de santé sexuelle en 1981, année d’émergence du sida, 22 % des HSH éliminaient des kystes d’E. *histolytica/dispar et* 4 % des kystes de *G. intestinalis.* Des larves de *S. stercoralis* étaient retrouvées chez 3,9 % d'entre eux [12]. Le premier cas de strongyloïdose symptomatique consécutif à une transmission sexuelle chez un HSH était rapporté en Californie en 1981 [14]. Parmi ses 12 contacts sexuels identifiés, 2 avaient une strongyloïdose asymptomatique. Les différents auteurs identifiaient la pratique de l'anulingus comme facteur indépendant de risque dans la transmission de parasites entériques dans cette population. Depuis la survenue de la pandémie du VIH, 19 cas, concernant tous chez des sujets séropositifs, ont été colligés dans la littérature de langue anglaise [7]. Trois étaient des cas isolés, dont un diagnostiqué à Paris [5], les autres appartenaient à de petites séries et provenaient du Brésil, du Venezuela et du Royaume-Uni. Une revue consacrée aux cas d'anguillulose diagnostiqués entre 2014 et 2019 dans six hôpitaux universitaires Français métropolitains a colligé 75 patients [9]. Parmi ceux-ci, neuf étaient retenus comme non-tropicaux, sans antécédents de voyage en zone tropicale. Cinq étaient des hommes ayant des rapports sexuels avec des hommes, trois avaient une infection VIH bien contrôlée. Les deux autres avaient des antécédents d'infections sexuelles multiples. Les quatre patients restants étaient des hétérosexuels, originaires d’Europe de l’Est vivant dans des conditions précaires.

La strongyloïdose est une helminthiase rencontrée dans les zones tropicales et tempérées chaudes. Elle a plusieurs originalités : existence d'un cycle d'auto-infestation interne qui explique sa longévité, fréquence des formes asymptomatiques, possibilité de formes graves sur certains terrains (traitement corticoïde prolongé, HTLV1), difficultés du diagnostic biologique, bonne réponse au traitement par ivermectine en l'absence d'immunosuppression [6]. Habituellement, la transmission se fait par pénétration transcutanée des larves, mais une contamination par voie muqueuse est possible [6]. Dans le cas des HSH, la contamination se ferait par transmission bucco-anale. Elle pourrait se faire également par pénétration des larves au niveau des muqueuses ou de la peau du pénis lors d'un rapport anal [1]. Cette helminthiase, lorsqu'elle est symptomatique, évolue de façon chronique, avec épisodes récurrents digestifs, cutanés ou pulmonaires. Chez deux de nos patients, l'infection a été découverte par un épisode aigu de primo-invasion (Observations 1 et 2) : douleurs abdominales et éruption cutanée, épisodes diarrhéiques accompagnés d’éosinophile élevée. Ces manifestations de strongyloïdose aiguë ont été rarement reportées [3]. Dans les observations 1 et 2, l’éosinophilie initiale était particulièrement élevée correspondant à une phase d'invasion récente.

Plusieurs facteurs associés ont été identifiés dans les publications rapportant des strongyloïdoses chez des HSH [7]. La parasitose est significativement associée à une infection VIH (incluant un taux bas de CD4 et l'absence de traitement antiviral), à des antécédents d'une ou plusieurs IST concomitantes *(C. trachomatis, Neisseria gonor-rhoeae, Treponema pallidum*, VHC), de protozoose *(E. histolytica, G. intestinalis)*, de voyage dans une zone endémique, de contacts sexuels oraux-génitaux-anaux, de nombreux partenaires provenant de zones endémiques, de pratique de *chemsex.* Nos patients présentaient ce profil avec plusieurs des facteurs associés identifiés. À notre connaissance, c'est la première fois dans la littérature que des pratiques de coprophagie sont rapportées (dans deux des quatre cas de notre série) comme élément unique ou prépondérant de la transmission de S. *stercoralis* en contexte sexuel HSH. Même si cette association entre une pratique à risque de contamination parasitaire (la coprophagie) ne repose que sur l'interrogatoire rétrospectif. Il semble qu'aucune question ne soit posée sur ces pratiques en pratique clinique, y compris devant des cas de parasitose digestive inexpliquée. Dans le cas n°4 retrouvé à l'interrogatoire, cette pratique était la seule avec l'anulingus évoquée lors du rapport sexuel précédent la strongyloïdose aiguë. Elle n'est pas citée dans la revue récente de Chessel *et al.* [7]. Dans une autre étude parisienne sur des cas probables de transmission sexuelle de strongyloïdose autochtone, cet élément ne figure pas dans l'interrogatoire [9]. Il est possible que pour des questions d'inoculum cette pratique, sur laquelle aucune question n'est posée et qui est rarement rapportée, soit un facteur bien plus favorisant que les simples rapports oraux-génitaux-anaux dont la fréquence est importante chez les HSH [2, 10]. Des cas de toxocarose par suite de pratiques de coprophagie ont été rapportés mais dans le spectre autistique [13].

Notre revue a l'inconvénient d’être rétrospective.

Les interrogatoires n'ont peut-être pas été assez bien dirigés chez ces patients présentant une pathologie à laquelle nous n'avions pas été confrontés auparavant chez des HSH. Dans deux cas, ils n'ont pas été poussés jusqu'aux pratiques copropha-giques dont la fréquence dans cette population n'est pas à notre connaissance documentée. Les enquêtes portant sur les comportements sexuels chez les HSH se sont surtout polarisées sur le sexe anal, oral-génital-anal et peu ou pas sur les autres pratiques, supposées être à peu de risque dans la transmission du VIH mais non dans celle d'autres IST. Il est important de connaître tous ces comportements afin de mener un interrogatoire adapté et proposer des mesures prophylactiques. En France, une étude a porté sur l'association entre les types de rencontre pour trouver un partenaire, les relations anales non protégées, un engagement dans un *sex group*, le risque d'infection à VIH et autres IST, chez des HSH [2]. Ce sont les rencontres dont le but principal était d'avoir des relations sexuelles qui se sont montrées comme comportements à risque. La réduction des attitudes à risque repose sur des stratégies basées sur des campagnes de prophylaxie pré-exposition. En Australie, 1 596 HSH ont été interrogés sur leurs pratiques sexuelles : s'embrasser était la plus commune, suivie par le sexe oral administré (86 %) ou reçu (83,9 %) [10]. L'anulingus actif était le moins pratiqué (38,7 %). Des différences étaient retrouvées selon les âges quand les partenaires étaient occasionnels. Ce type d’étude devrait être poursuivi pour mieux cerner les sous-populations à risque.

L'un des problèmes que nous avons rencontrés est la quasi-impossibilité à tracer et à prendre en charge les cas contacts et le refus de certains patients à répondre à un questionnaire intrusif. Nos patients ont eu des relations multiples avec des sujets anonymes rencontrés dans des lieux de rencontre gays. Deux hommes immigrés récents, originaires d’Afrique subsaharienne, faisaient partie du *sex group* d'un de nos patients depuis deux ans. En situation irrégulière, ils n'ont pas souhaité se faire dépister.

Il est probable que la strongyloïdose à transmission sexuelle est sous-estimée chez les HSH. C'est une helminthiase asymptomatique dans environ 50 % des cas comme en témoignent deux de nos observations. Le diagnostic biologique est difficile. L’éosinophilie, habituellement fluctuante dans cette parasitose, peut être absente. Les examens parasitologiques des selles (examen direct et concentration) sous souvent mis en défaut. La méthode d'extraction des larves de Baermann a une sensibilité de 40 à 80 %, nettement supérieure aux examens classiques. Mais elle est chronophage, mise en œuvre seulement par des laboratoires spécialisés. Un grand progrès a été apporté par l'arrivée de méthodes sérologiques disponibles dans le commerce dont la sensibilité varie de 75,4 % à 94,7 % et la spécificité de 42,1 % à 95,4 % [6]. La détection d’ADN parasitaire dans les selles n'est pratiquée que par des laboratoires de référence.

La strongyloïdose devrait être reconnue comme IST chez les HSH. Les médecins, même spécialistes, exerçant en métropole connaissent peu ou pas le risque d'infection à S. *stercoralis* chez des homosexuels n'ayant jamais voyagé sous les tropiques. La possibilité de contracter cette parasitose à la suite de pratiques bucco-anales ou coprophagiques doit être diffusée auprès des associations de lutte contre le VIH/sida. Les mesures prophylactiques sont similaires à celles préconisées dans les autres infections entériques chez les HSH [1, 8]. Les mesures recommandées sont tout d'abord les mesures d'hygiène basique : lavage des mains après avoir eu un contact avec l'anus ou le rectum de son partenaire, utilisation de digue dentaire pour la pratique de l'anulingus, changement de préservatif entre sexe anal et buccal. Une modification des comportements sexuels est à envisager. Elle vise à diminuer les actes favorisant une contamination fécale, à proscrire le *chemsex*, pour atteindre une pratique sexuelle sans risque.

## Conclusion

La strongyloïdose, géohelminthiase intestinale des zones tropicales, a été décrite en dehors des zones endémiques, comme la France métropolitaine, chez des hommes ayant des relations sexuelles avec des hommes, favorisée par des contacts oraux-génitaux-anaux. Elle doit être évoquée devant une éosinophilie isolée accompagnée ou non de signes digestifs même chez des sujets n'ayant jamais voyagé en milieu tropical. L'interrogatoire doit rechercher s‘il y a eu des contacts oraux-génitaux-anaux ou des pratiques coprophagiques avec des partenaires originaires de pays tropicaux. Chez des patients déjà étudiés, on relève la fréquence des antécédents d’IST diverses et de la pratique du *chemsex.* Un interrogatoire détaillé permettra d'identifier les sujets à risque qui seraient justiciables d'un traitement systématique par ivermectine même en l'absence de signes cliniques et d'hyperéosinophilie.

## Sources de financement

Aucune.

## Considérations éthiques

Tous les sujets concernés ont donné leur consentement.

## Contribution des auteurs

Michel Develoux : conception, recueil des observations, rédaction

Martin Siguier : recueil des observations, rédaction Christophe Hennequin : analyses parasitologiques Gilles Pialoux : recueil des observations, rédaction, validation

## Conflit d'intérêt

Les auteurs ne déclarent aucun conflit d'intérêt.
