# Supplementary material for: Sexual transmission of strongyloidiasis in men who have sex with men (MSM) in Paris
Source: Med Trop Sante Int. 2025 Mar 5;5(1):mtsi.v5i1.2025.660. [Article in French] doi: 10.48327/mtsi.v5i1.2025.660 (PMC12001986; doi:10.48327/mtsi.v5i1.2025.660)
Supplement: Related texts

# Research article: Sexual transmission of strongyloidiasis in men who have sex with men (MSM) in Paris

## Introduction

Sexual transmission of intestinal parasites among men who have sex with men (MSM) has long been reported [12, 14]. These are mainly protozoa *(Entamoeba histolytica* and *Giardia intestinalis).* The practice of rimming, which facilitates the fecal-oral transmission of enteric parasites, is presented as the main risk factor predicting this type of transmission. Strongyloidiasis has been very rarely described in this context and does not appear in recent general reviews of emerging or re-emerging sexually transmitted infections (STIs) [4, 15]. We present four cases of *Strongyloides stercoralis* infection in MSM associated with specific sexual practices in individuals living in mainland France who had not traveled to endemic areas.

## Patients

### Observation 1

A 36-year-old man consulted the Infectious and Tropical Diseases Department of the Tenon Hospital in Paris in 2012 for periumbilical pain and hypereosinophilia at 5,720/m^3^. He is an MSM and his HIV infection was detected six years ago. He is treated with Truvada/Prezista/Norvir, the viral load was undetectable and the CD4 count was 358/mm^3^. The patient had a history of multiple STIs: multiple episodes of syphilis, hepatitis C, and genital warts.

Symptoms began 10 days before presentation with abdominal pain and diarrhea of sudden onset. Five days later, a urticarial rash appeared on the buttocks, which disappeared after six days. *Strongyloides stercoralis* larvae were found by direct examination of the stool, as well as by concentration and the Baermann method. Serology for strongyloidiasis and other helminthiases was negative. The patient was treated with ivermectin 200 µg/kg for two days. The clinical signs resolved rapidly. One month later, the hypereosinophilia had disappeared and the stool test was negative. The patient had traveled to Morocco six years earlier, the only time he had left France. He had a regular partner whose parasitologic tests were negative. He frequented a gay sauna where he had numerous unprotected sexual encounters outside the HIV risk (undetectable viral load) with men, some of whom were from areas endemic for strongyloidiasis. It was not possible to administer a detailed questionnaire about his sexual practices.

### Observation 2

A 64-year-old man with known HIV infection was hospitalized in 2018 because of diarrhea of one month duration with abdominal pain, generalized pruritus, and high eosinophilia (9,330/ mm^3^). He self-identified as bisexual. HIV infection had been diagnosed 12 years earlier and he was treated with abacavir/dolutegravir/lamivudine (undetectable viral load, CD4: 779/µL). He had regular follow-up with blood counts twice a year. There was no evidence of hypereosino-philia before the diarrheal episode. The patient's medical history included numerous sexually transmitted infections: *Chlamydia trachomatis*, hepatitis A and C. There was mention of a brief stay in sub-Saharan Africa four years prior to hospitalization. Hypereosinophilia was confirmed by a second sample. Strongyloidiasis serology was positive, and larvae were found only by the Baermann method of extraction. He received two doses of ivermectin (200 µg/kg) one day apart. Clinical signs resolved rapidly. One month later, the eosinophilia had normalized and the stool parasitologic examination was negative. It was not possible to propose a detailed questionnaire on his sexual practices.

### Observation 3

A 52-year-old MSM being treated in the infectious diseases department for HIV infection was referred in 2018 for evaluation of hypereosinophilia (2,663/mm3), which was incidentally detected 1 year earlier and confirmed 6 (1,664/mm3) and 12 months later (1,840/mm3). HIV infection had been diagnosed 19 years earlier and he was receiving antiretroviral treatment with dolutegravir/abacavir/lamivudine (undetectable viral load, CD4 > 900/µL). His history was marked by several episodes of STI: genital syphilitic chancre, pharyngeal gonorrhea, self-limiting hepatitis C. He had never left France, lived with one partner but had other partners. He had regular unprotected “group sex” with 12 to 14 partners per month. The group practiced rimming and engaged in chemsex (the practice of combining sex with drug use). Two migrants from sub-Saharan Africa had joined the group two years earlier. The patient tested positive for strongyloidiasis, and larvae were found on direct examination and formalin ether concentration. A single course of ivermectin proved insufficient, and cure was achieved with the same dose on two consecutive days. The partner's parasitologic tests were negative; the two people, who had migrated from sub-Saharan Africa, did not want to be tested. Continuing the questioning of this patient's sexual practices, we found, in addition to rimming, insertive but not receptive fistfuck without gloves and scatophilia with receptive coprophagy.

### Observation 4

A 52-year-old MSM with HIV infection was hospitalized for evaluation of asymptomatic hypereosinophilia (2,673/mm^3^) detected during a routine check-up of his infection and confirmed by a check-up two months later (1,533/mm3). He had never traveled outside mainland France. HIV infection had been detected 28 years earlier. He was treated with Truvada and Isentress (negative viral load, CD4: 861). His medical history included several sexually transmitted infections: secondary syphilis, anorectal gonorrhea, genital warts. He had numerous partners and practiced chemsex. Clinical examination was normal. Larvae of *Strongyloides stercoralis* were found by direct examination and concentration methods, serology was positive. He was successfully treated with ivermectin. Resuming the questioning about his sexual practices, we found that in addition to rimming, this patient had engaged in a single episode of scatophiliac practices with receptive coprophagy two months before the screening for hypereosinophilia, after a meeting someone on an MSM site.

## Discussion

Sexually transmitted enteric infections favored by certain practices have been reported in MSM [4, 15]. These can be of bacterial, viral or parasitic origin. The consequences can be worrying, as demonstrated by the spread of multidrug-resistant shigellosis in different continents [8]. The sexual transmission of enteric parasites among MSM has been known since before the advent of HIV/AIDS [12, 14]. These are mainly protozoa *(Entamoeba histolytica, Giardia intestinalis, Cryptosporidium* sp.) [1, 4, 14, 15]. Transmission of helminths *(Enterobius vermicularis, Strongyloides stercoralis)* is less common [7, 11]. In a study conducted in a sexual health clinic in 1981, the year of the emergence of AIDS, 22% of MSM had *E. histolytica/dispar* cysts and 4% had *G. intestinalis* cysts. S. *stercoralis* larvae were found in 3.9% of them [12]. The first case of symptomatic strongyloidiasis after sexual transmission in an MSM was reported in California in 1981 [14]. Among his 12 identified sexual contacts, 2 had asymptomatic strongyloidiasis. The various authors identified the practice of rimming as an independent risk factor for intestinal parasite transmission in this population. Since the onset of the HIV pandemic, 19 cases have been reported in the English-language literature, all in HIVpositive individuals [7]. Three were isolated cases, one of which was diagnosed in Paris [5], and the others were small series from Brazil, Venezuela, and the United Kingdom. A review of cases of anguillulosis diagnosed between 2014 and 2019 in six French metropolitan university hospitals collected 75 patients [9]. Of these, nine were considered non-tropical, with no history of travel to tropical areas. Five were men who have sex with men, three had well-controlled HIV infection. The other two had a history of multiple sexual infections. The remaining four patients were heterosexual, from Eastern Europe and living in precarious conditions.

Strongyloidiasis is a helminthiasis found in tropical and warm temperate zones. It has several unique features: the existence of a cycle of internal autoinfection, which explains its longevity, the frequency of asymptomatic forms, the possibility of severe forms in certain cases (prolonged corticoid treatment, HTLV1), difficulties in biological diagnosis, good response to treatment with ivermectin in the absence of immunosuppression [6]. Transmission is usually by transcutaneous penetration of the larvae, but mucosal contamination is also possible [6]. In the case of MSM, contamination occurs by oral-anal transmission. It can also occur by penetration of the larvae into the mucosa or skin of the penis during anal intercourse [1]. When symptomatic, this helminthiasis is chronic, with recurrent gastrointestinal, cutaneous, or pulmonary episodes. In two of our patients, the infection was discovered by an acute episode of primary invasion (observations 1 and 2): abdominal pain and skin rash, diarrheal episodes accompanied by high eosinophilia. These manifestations of acute strongyloidiasis have rarely been reported [3]. In observations 1 and 2, the initial eosinophilia was particularly high, corresponding to a recent invasion phase. Several associated factors have been identified in publications reporting strongyloidiasis in MSM [7]. The parasitosis is significantly associated with HIV infection (including low CD4 count and absence of antiviral treatment), history of one or more concomitant STIs *(C. trachomatis, Neisseria gonorrhoeae, Treponema pallidum*, HCV), protozoan infection *(E. histolytica, G. intestinalis)*, travel to an endemic area, oral-genital-anal sexual contact, numerous partners from endemic areas, and chemsex. Our patients presented this profile with several of the identified associated factors. To our knowledge, this is the first time in the literature that coprophagic practices have been reported (in two of the four cases in our series) as a unique or predominant element of transmission of *S. stercoralis* in the MSM sexual context. Even if this association with a practice at risk of parasitic contamination (coprophagy) is based only on retrospective questioning. No questions seem to be asked about these practicesin clinical practice, even in cases of unexplained digestive parasitosis. In case no. 4, this practice was the only one reported, along with anilingus, during the sexual intercourse preceding the acute strongyloidiasis. It is not cited in the recent review by Chessel *et al.* [7]. In another Paris study of probable cases of sexual transmission of autochthonous strongyloidiasis, this element is not included in the questionnaire [9]. It is possible that for inoculum issues this practice, about which no questions are asked and which is rarely reported, is a much more favorable factor than simple oral-genital-anal intercourse, which is common among MSM [2, 10]. Cases of toxocariasis as a result of coprophagic practices have been reported, but in the autistic spectrum [13].

Our review has the disadvantage of being retrospective. The interviews may not have been well targeted in these patients, who presented a pathology that we had not previously encountered in MSM. In two cases, they did not go so far as to ask about coprophagy, the frequency of which, to our knowledge, has not been documented in this population. Surveys of sexual behavior among MSM have focused mainly on anal, oral-genital-anal sex and little or nothing on other practices that are thought to carry low risk of HIV transmission but no other STIs. It is important to be aware of all these behaviors in order to conduct appropriate counseling and propose prophylactic measures. In France, a study examined the association between the type of encounter used to find a partner, unprotected anal intercourse, participation in a sex group, and the risk of HIV and other STI infection among MSM [2]. It was the encounters whose primary purpose was to have sex that were found to be risky behaviors. Strategies based on pre-exposure prophylaxis campaigns have been used to reduce risky behavior. In Australia, 1,596 MSM were asked about their sexual practices: kissing was the most common, followed by oral sex given (86%) or received (83.9%) [10]. Active rimming was the least common practice (38.7%). Differences were found by age when partners were casual. This type of study should be continued to better identify subpopulations at risk.

One of the problems we encountered is the near impossibility of tracking and managing contact cases, and the refusal of some patients to answer an intrusive questionnaire. Our patients had multiple relationships with anonymous individuals they met in gay venues. Two recent immigrants from sub-Saharan Africa had been part of one of our patients’ sex group for two years. Because they were in an irregular situation, they did not want to be tested.

It is likely that sexually transmitted strongyloidiasis is underestimated in MSM. It is an asymptomatic helminthiasis in about 50% of cases, as evidenced by two of our observations. The biological diagnosis is difficult. Eosinophilia, which is usually variable in this parasitic disease, may be absent. Parasitologic stool examinations (direct examination and concentration) are often deficient. The Baermann larval extraction method has a sensitivity of 40-80%, significantly higher than conventional tests. However, it is time-consuming and can only be performed by specialized laboratories. Great strides have been made with the advent of commercially available serologic methods with sensitivities ranging from 75.4% to 94.7% and specificities ranging from 42.1% to 95.4% [6]. Detection of parasite DNA in stools is performed only by reference laboratories. Strongyloidiasis should be recognized as an STI in MSM.

Doctors, even specialists, practicing in mainland France are unaware or unaware of the risk of S. *stercoralis* infection in homosexuals who have never traveled to the tropics. The possibility of contracting this parasitic disease as a result of oral-anal or coprophagic practices must be disseminated to HIV/AIDS associations. Prophylactic measures are similar to those recommended for other enteric infections in MSM [1, 8]. The recommended measures are primarily basic hygienic measures: washing hands after contact with a partner's anus or rectum, using a dental dam for rimming, changing condoms between anal and oral sex. Modification of sexual behavior should be considered. The goal is to reduce activities that promote fecal contamination, to prohibit chemsex, and to achieve safe sex.

## Conclusion

Strongyloidiasis, an intestinal geohelminthiasis of tropical areas, has been described outside endemic areas, such as metropolitan France, in men who have sex with men, favored by oral-genital-anal contact. It should be considered in the case of isolated eosinophilia, with or without digestive signs, even in subjects who have never traveled to tropical areas. Questioning should include whether there has been oral-genital-anal contact or coprophagous practices with partners from tropical countries. In patients who have already been examined, the frequency of a history of various sexually transmitted diseases and the practice of chemsex is noted. A detailed interview will make it possible to identify persons at risk who would be suitable for systematic treatment with ivermectin even in the absence of clinical signs and hypereosinophilia.

## Funding

None.

## Ethical considerations

All subjects gave informed consent.

## Authors’ contributions

Michel Develoux: design, collection of observations, writing

Martin Siguier: collection of observations, writing Christophe Hennequin: parasitological analyses Gilles Pialoux: collection of observations, writing, validation

## Conflicts of interest

The authors declare no conflict of interests.
